# Disparities in ratings of internal and external applicants: A case for model-based inter-rater reliability

**DOI:** 10.1371/journal.pone.0203002

**Published:** 2018-10-05

**Authors:** Patrícia Martinková, Dan Goldhaber, Elena Erosheva

**Affiliations:** 1 Department of Statistical Modelling, Institute of Computer Science of the Czech Academy of Sciences, Prague, Czech Republic; 2 Institute for Research and Development of Education, Faculty of Education, Charles University, Prague, Czech Republic; 3 Center for Education Data and Research, School of Social Work, and the Center for Statistics in the Social Sciences, University of Washington, Seattle, WA, United States of America; 4 Department of Statistics, School of Social Work, and the Center for Statistics in the Social Sciences, University of Washington, Seattle, WA, United States of America; 5 Laboratoire J.A. Dieudonné, Université Côte d’Azur, CNRS, Nice, France; Charles P. Darby Children's Research Institute, 173 Ashley Avenue, Charleston, SC 29425, USA, UNITED STATES

## Abstract

Ratings are present in many areas of assessment including peer review of research proposals and journal articles, teacher observations, university admissions and selection of new hires. One feature present in any rating process with multiple raters is that different raters often assign different scores to the same assessee, with the potential for bias and inconsistencies related to rater or assessee covariates. This paper analyzes disparities in ratings of internal and external applicants to teaching positions using applicant data from Spokane Public Schools. We first test for biases in rating while accounting for measures of teacher applicant qualifications and quality. Then, we develop model-based inter-rater reliability (IRR) estimates that allow us to account for various sources of measurement error, the hierarchical structure of the data, and to test whether covariates, such as applicant status, moderate IRR. We find that applicants external to the district receive lower ratings for job applications compared to internal applicants. This gap in ratings remains significant even after including measures of qualifications and quality such as experience, state licensure scores, or estimated teacher value added. With model-based IRR, we further show that consistency between raters is significantly lower when rating external applicants. We conclude the paper by discussing policy implications and possible applications of our model-based IRR estimate for hiring and selection practices in and out of the teacher labor market.

## Introduction

Ratings have been part of the assessment landscape in many areas for many years. They are considered the gold standard of science and are present in peer review of grant proposals or journal articles [1], are integral parts of educational and psychological assessments [2], and are present in student admission processes [3] or selection of new hires. The legitimacy of rating procedures depends crucially on the reliability, validity and fairness of ratings systems and processes [4].

There are numerous covariates that may affect ratings, such as an applicant’s or reviewer’s gender, ethnicity and major or research area [1]. These factors may be potential source of bias and unfairness in ratings, but may also influence the inter-rater reliability (IRR) [5]. One factor that may cause bias is institutional proximity of the applicant. Such “affiliation bias” has, for instance, been shown in grant proposal peer reviews [6–8].

In labor economics, both theoretical and empirical studies confirm the commonsense notion that the human resource management processes used to make hiring decisions can have profound effects on the workforce labor productivity [9–11]. The productivity of new hires is dependent both on their individual attributes and the fit between employees and organizations [12–13]; social competency, compatibility, and capital may be highly valuable and support positive work environments, productivity, and success of organizations as a whole.

In many contexts, the selection of an employee for a position can come down to a choice between an external applicant and an insider, i.e. an applicant that is internal to a firm or organization. Yet there is relatively little evidence on how hiring processes treat external and insider applicants.

Studies that focus on internal (promotions or lateral transfers) and external hiring find that external candidates face an uphill battle to be hired over internal candidates, in that they tend to need better observable indicators of quality than their internal peers [13–14]. This finding may be related to hiring managers having relatively more knowledge about internal candidates, the importance of firm-specific human capital, or the desire by firms to create promotion-related incentives for other employees [15].

One important issue that has received little attention is whether the applicant selection tools and ratings often used in assessing job applicants function differently for internal and external applicants. In particular, internal applicants may have advantages over external applicants, due to their knowledge of the attributes that employers are looking for, because they are more likely to receive recommendations from individuals who understand the attributes that employees are looking for, or because they are directly known by hiring officials.

In this paper, we examine how the ratings on applicant selection tools compare for internal and external applicants to teaching positions in Spokane Public Schools (SPS), a relatively large school district in eastern Washington State. We use mixed-effect models [16] allowing rater- and applicant- covariates to test for bias. We analyze differences in ratings between external and internal applicants, with a particular focus on variance and IRR for these groups. We also derive a test of between-group differences in IRR, relying on mixed-effect models allowing group-specific variance terms of random variables.

### SPS teacher applicant selection tool

For hiring decisions, SPS utilizes a four-stage hiring process [17]. In the first step, an online application management system is used for uptake and initial check of applications. Next, pre-screening of potential applicants is made by central office human resources officials. In the third stage, screening of applicants meeting initial screening standards is done by school-level hiring officials. Finally, applicants with the highest school-level screening scores are invited for in-person interviews: job offers are made based upon judgments after this final stage.

In this work, we analyze data from school-level screening, the third stage of the SPS hiring process. Important for our purposes, a large number of applicants who are screened at this stage have multiple ratings. Applicants at this stage (for the majority of the study period) were rated on a 6-point scale in nine criteria, each a subcomponent of the rating instrument. The screening rubric (which is on a 54-point scale) and criteria are outlined in Table 1. Ratings were based on written materials that were included in the application and in supporting documentation (e.g., resume, cover letter, and at least three letters of recommendation). A summative score was used to select which candidates receive in-person interviews. During the study period, about 40% of applicants screened on the school level were not advanced to an interview. In previous studies, both the district-level and school-level selection tools have been shown to be predictive of later teacher and student outcomes [17].

**Table 1:** 54-point screening rubric.

Criterion	Look for …
**Certificate and Education**	Note completion of course of study, certificate held (current or pending), and education.
**Training**	Look for quality, depth, and level of candidate’s additional training related to position.
**Experience**	Note the degree to which experience supports the prediction of success, not just the number of years. A beginning candidate could be rated highly.
**Classroom Management**	Look for specific references to successful strategies. This may mean *planned and directed* rather than *quiet and orderly*. Effectively handles large/small or ethnically/sociologically diverse groups; develops routines and procedures to increase learning; establishes clear parameters; and responds appropriately.
**Flexibility**	Note multiple endorsements, activity, coaching interests, student, building or district, or community support. Willing to learn new concepts and procedures; successfully teachers a variety of assignments; effectively uses various teaching styles.
**Instructional Skills**	Look for specific references in support of skill in this area: plans; implements; evaluates; relates to students; creative; employs multiple approaches; monitors and adjusts; uses culturally responsive strategies appropriate to age, background, and intended learning of students.
**Interpersonal Skills**	Develops and maintains effective working relationships with diverse staff, students, parents/guardians, and community.
**Cultural Competency**	Look for specific references to successful strategies for building and maintaining a relationship with each student and their family. This may not be explicitly mentioned, but the following strategies offer some evidence of cultural competency: specific instructional strategies providing each student access to a rigorous curriculum, inclusive/respectful language about students and families, a belief that all children can achieve at high levels, mention of conflict resolution/restorative practices, specific instructional strategies for integrating culturally responsive materials that are also rigorous, and appropriate statements about their work with diverse populations. Note relevant training, coursework, and authors/book titles listed.
**Preferred Qualifications**	Look for possession of qualifications as indicated on the job posting.

### Research questions

We analyze rating disparities of internal and external applicants. Specifically, we address the following research questions

1. Do external applicants receive lower ratings on subcomponents and in total than internal applicants?
2. Are any differences in ratings between the two groups explainable by other measures of applicant qualifications and quality, available before hiring decision (e.g. years of experience, licensure test scores), or measured in years following after the hiring decision (e.g. estimated teacher value added to subsequent achievement of their students)?
3. Does the magnitude of variance components differ for internal versus external applicants?
4. Is the IRR equal for internal and external applicants, or is it higher for insiders?

## Methods

### Teacher application dataset

Our dataset contains ratings of applicants (assessees) for teaching positions in SPS during the school years 2008–09 through 2012–13. This includes a total of 3,474 individual ratings with known applicant and rater ID and job location, representing 1,090 individual applicants rated by 137 raters for classroom-teaching job postings at 54 job locations (schools). These units are partially crossed both with applicants (many applicants applied to multiple schools) and with raters (some raters rated for multiple schools).

Applicants were rated on a 6-point scale in nine subcomponents (Table 1), and the summative score (on a cumulative 54-point scale) was also provided. Multiple ratings of the same applicant may occur within the same school during one time period (e.g. some schools employ more raters and use average total score to rank the applicants), based on multiple applications to the school at one time (to multiple job openings) or over time, and/or across different schools in the district.

We also consider three other proxies of applicant quality and qualifications: teaching experience (in years), state licensure scores (WEST-B average, math, reading and writing, all standardized statewide) and, for applicants hired in Washington State, estimates of teacher value added to achievement of their students in mathematics and reading. Teacher value added, in simple terms, is the estimated contribution of teachers toward student achievement gains on standardized tests, generally adjusted for student background characteristics, such as free or reduced-price lunch status. The specific linear model used to generate the value added we used in this paper is described elsewhere [17].

We consider an applicant to be internal when he or she either was previously employed as a teacher in the district (e.g., at a different school, different position or in a different time period) or had completed his or her student teaching (part of teacher training) in the district. Otherwise, the applicant is considered to be external to the district at the time she/he is rated. Of all ratings, 2,322 were for internal applicants, and 1,152 were for external applicants. Fifty-one applicants were, by our criteria, marked as external for some ratings and as internal for others. We keep these individuals in the analysis. For comparison of the two samples, they are included in both pools depending on the status when measure was taken. In the analyses, applicant status is included in the model.

### Data analysis

Statistical environment R version 3.4.3 [18] and its libraries lme4 [19–20] and lmerTest [21] are used for analyses as specified in subsections below. Library data.table [22] is used to reshape the data, and library ggplot2 [23] is used to prepare graphics. Commented sample R code is provided in supplemental materials.

#### Absolute differences in summative ratings of external and internal applicants

Descriptive statistics for all measures are calculated for internal and external applicants. Two sample t tests are used to test significance of the differences, and we utilize the Benjamini–Hochberg correction of p values to account for multiple comparisons [24]. Besides p values, Cohen’s *d*, defined as the absolute difference between means of the two groups divided by a standard deviation for the data [25], is used to evaluate effect sizes of the differences.

We begin testing for bias in total ratings with respect to applicant internal/external status in Model (1):

$$Y_{ijl}=\mu +\beta_{0}\omega_{i}+A_{i}+B_{j}+S_{l}+AS_{il}+e_{ijl}$$ (1)

In this model, *μ* is the mean for external applicants, *β*_0_ is the estimated effect of being an internal applicant (identified by *ω*_*i*_ = 1). We also assume random effects for applicant *A*_*i*_, rater *B*_*j*_, and school *S*_*l*_ to account for the hierarchical structure of the data, and we include applicant-school interactions *AS*_*il*_ to account for the possibility of applicant-school matching effects. The residual *e*_*ijl*_ reflects the departure of observed scores on the rating of applicant *i* by rater *j* for school *l* from what would be expected given the grand mean, the individual’s true score, and the effect of the rater, school and applicant-school interaction. Residual includes a possible interaction between applicant and rater and between rater and school, which are not included in the model since the data contains limited multiple ratings of the same applicant by the same rater and limited ratings of the same rater for different schools. We assume joint normal, uncorrelated and mean-zero distributions for applicants, raters, and residuals. In additional models, we further add fixed effects ***β*** describing the *i*^th^ teacher’s qualities ***x***_*i*_: number of years of experience, licensure scores (WEST-B) as well as estimate of teacher value added to subsequent achievement of their students in mathematics and reading in the subpopulation of teachers hired in Washington State:

$$Y_{ijl}=\mu +\beta_{0}\omega_{i}+\beta^{T}x_{i}+A_{i}+B_{j}+S_{l}+{AS}_{il}+e_{ijl}$$

In all models, we test for significance of applicant internal status *β*_0_ using likelihood ratio tests [26].

#### Variance decomposition and testing for differential IRR for internal and external applicants

Starting with Model defined by Eq 1 we estimate the contributions of variance from the various sources: the applicant effect, the rater effect, the school effect, applicant-school matching effects, and the residual:

$$\sigma_{Y}^{2}=\sigma_{A}^{2}+\sigma_{B}^{2}+\sigma_{S}^{2}+\sigma_{AS}^{2}+\sigma_{e}^{2}.$$

Assuming single raters, inter-rater reliability of applicant ratings within schools is defined as ratio of true-score variance to total variance

$$IRR_{within}=\frac{\sigma_{A}^{2}+\sigma_{S}^{2}+\sigma_{AS}^{2}}{\sigma_{Y}^{2}}=\frac{\sigma_{A}^{2}+\sigma_{S}^{2}+\sigma_{AS}^{2}}{\sigma_{A}^{2}+\sigma_{B}^{2}+\sigma_{S}^{2}+\sigma_{AS}^{2}+\sigma_{e}^{2}}.$$ (2)

It is clear from Eq 2 that IRR is higher when applicants, schools and applicant-school interactions account for substantial proportion of rating variation and raters and other sources of variation do not.

When analyzing between-group differences in reliability, IRR is usually calculated separately for groups using stratified data [27, 28]. We take a more flexible approach to test for differential IRR by group. Specifically, in the following model we allow variance terms of main random effects to differ by group (i.e. for internal and external applicants):

$$Y_{ijl}=\mu +\omega_{i}\beta_{0}+(1-\omega_{i})A_{0i}+\omega_{i}A_{1i}+\left(1-\omega_{i}\right)B_{0i}+\omega_{i}B_{1i}+\left(1-\omega_{i}\right)S_{0l}+\omega_{i}S_{1l}+AS_{il}+e_{ijl}.$$ (3)

In model defined by Eq 3 (also addressed as Model (3) below), estimates of variance components are obtained for internal and for external applicants. IRR is then estimated using Eq 2 for the two sets of variance component estimates. The total variance now decomposes into 8 terms, $\sigma_{0A}^{2},\;\sigma_{1A}^{2},\;\sigma_{0B}^{2},\;\sigma_{1B}^{2},\;\sigma_{0S}^{2},\;\sigma_{1S}^{2},\;\sigma_{AS}^{2},\sigma_{e}^{2},$ and the within-school IRR now varies for the two groups due to variance components that are allowed to vary by group:

$$IRR_{within,ext}=\frac{\sigma_{0A}^{2}+\sigma_{0S}^{2}+\sigma_{AS}^{2}}{\sigma_{0A}^{2}+\sigma_{0B}^{2}+\sigma_{0S}^{2}+\sigma_{AS}^{2}+\sigma_{e}^{2}},$$ (4)

$$IRR_{within,int}=\frac{\sigma_{1A}^{2}+\sigma_{1S}^{2}+\sigma_{AS}^{2}}{\sigma_{1A}^{2}+\sigma_{1B}^{2}+\sigma_{1S}^{2}+\sigma_{AS}^{2}+\sigma_{e}^{2}}.$$ (5)

We use bootstrap procedures to calculate confidence intervals for the IRR estimates and to calculate confidence intervals for the difference between the IRRs for internal and external applicants. All calculations are performed for the summative overall score as well as for individual subcomponents.

#### Effect of higher number of raters

We use the *prophecy formula* [29–30] and generalizability theory [31] to provide estimates of IRR using various potential scoring designs, i.e., assuming differing number of raters. IRR is estimated as the ratio of “true score” variance of applicant for a given school to the total variance of the average scores from multiple ratings (the true score plus the error variance of the average). For *J* raters, the average ratings is $\bar{Y}=\frac{\sum_{j=1}^{J}Y_{ijl}}{J},$ and the variance decomposes to

$$\sigma_{\bar{Y}}^{2}=\sigma_{A}^{2}+\sigma_{B}^{2}/J+\sigma_{S}^{2}+\sigma_{AS}^{2}+\sigma_{e}^{2}/J.$$

Higher number of raters *J* and lower error variance, $\frac{\sigma_{B}^{2}}{J}+\frac{\sigma_{e}^{2}}{J}$ implies higher within-school IRR:

$$IRR_{\bar{Y},within}=\frac{\sigma_{A}^{2}+\sigma_{S}^{2}+\sigma_{AS}^{2}}{\sigma_{\bar{Y}}^{2}}=\frac{\sigma_{A}^{2}+\sigma_{S}^{2}+\sigma_{AS}^{2}}{\sigma_{A}^{2}+\sigma_{B}^{2}/J+\sigma_{S}^{2}+\sigma_{AS}^{2}+\sigma_{e}^{2}/J}.$$ (6)

We provide estimates of IRR for internal and external applicants using Model (3) for cases of one, two and three raters. We also use the standard error of measures (SEM), the square root of $\frac{\sigma_{B}^{2}}{J}+\frac{\sigma_{e}^{2}}{J}$, to evaluate the precision of estimates of the score level.

To analyze whether the reliability of ratings influences their predictive validity, we examine correlations of ratings with estimates of teacher value added. Correlations between teacher value added and ratings are calculated from the full sample without accounting for applicant status. Correlations between teacher value added and average of two or three raters, are estimated using IRR estimates under Model (3) with respect to applicant status by employing the *attenuation formula* [32–33]:

$$corr\left(Y,Z\right)=\frac{cov\left(\mu +\varepsilon_{\mu },\theta +\varepsilon_{\theta }\right)}{\sqrt{var\left(\mu +\varepsilon_{\mu }\right)\;var\;\left(\theta +\varepsilon_{\theta }\right)}}=corr\left(\mu ,\theta \right)\sqrt{R_{\mu }}\sqrt{R_{\theta }}.$$ (7)

## Results

### Characteristics of internal and external applicants

Table 2 provides applicant pre-hiring characteristics, summative and sub-component ratings received by each applicant during the hiring process as well as applicant’s subsequent quality measures (estimated teacher value added). We observe a significantly higher male to female ratio and greater experience in external applicants. While licensure scores are more often missing in external applicants, and later value added estimates are less often available due to the lower hiring percentage in external applicants, the available mean licensure scores and mean value added estimates of internal and external applicants are comparable.

**Table 2:** Applicant characteristics for internal and external applicant ratings.

Characteristics		Internal				External				Effect size
		Obs.	N	Mean	SD	Obs.	N	Mean	SD	
Gender (Female ratio)		2257	644	0.77	0.42	1024	392	0.67	0.47	0.23***
Teaching experience		2322	678	3.35	4.87	1149	461	4.62	5.34	0.25***
WEST-B										
	Average	1056	251	-0.04	0.71	355	148	-0.11	0.75	0.10
	Math	1060	252	-0.04	1.09	355	148	-0.04	1.01	0.00
	Reading	1057	252	-0.08	0.89	355	148	-0.21	0.96	0.14
	Writing	1056	251	0.01	0.78	355	148	-0.09	0.89	0.12
54-Pt Rubric										
	Total	2322	678	39.13	6.63	1152	463	35.22	6.80	0.58***
	Certificate and Education	2226	668	5.13	0.80	1100	446	4.91	1.04	0.24***
	Training	2314	677	4.11	1.12	1137	460	3.56	1.18	0.48***
	Experience	2322	678	4.21	1.00	1151	463	3.77	1.09	0.42***
	Management	2301	676	4.22	0.94	1145	462	3.75	1.02	0.48***
	Flexibility	2313	678	4.37	0.92	1146	461	3.99	1.00	0.39***
	Instructional Skills	2316	678	4.34	0.98	1147	463	3.82	1.03	0.52***
	Interpersonal Skills	2310	678	4.52	0.86	1143	461	4.14	1.00	0.41***
	Cultural Competency	2302	677	4.12	0.93	1141	461	3.70	1.09	0.41***
	Preferred qualifications	1720	614	4.09	1.28	840	391	3.58	1.27	0.40***
Later VA										
	Math	271	83	-0.04	0.23	32	17	-0.05	0.14	0.05
	Read	279	83	-0.09	0.19	57	24	-0.06	0.15	0.15

Notes: WEST-B: scores on state licensure test, standardized statewide, VA: teacher value added estimates based on changes of student performance in achievement tests. Obs.: number of observations, N: number of applicants, SD: standard deviation, significance levels for p values corrected for multiple comparisons

* p < 0.05

** p < 0.01

*** p < 0.001.

### Differences in rating on the 54-point screening rubric

**Fig 1:**
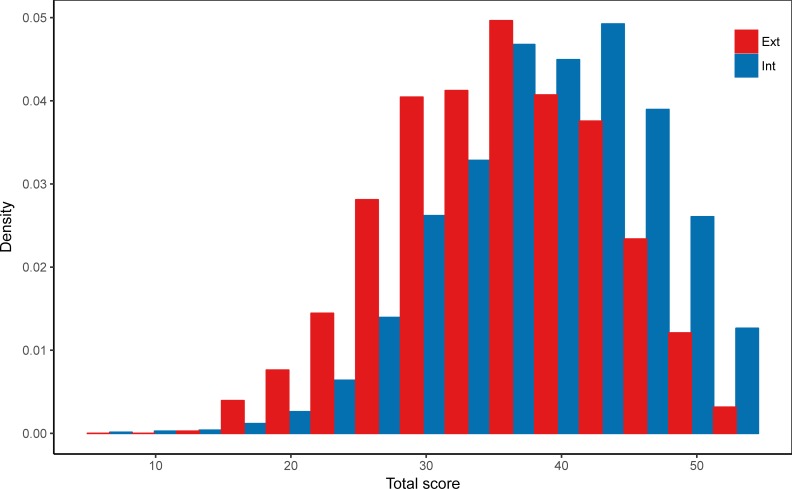
Distribution of total ratings for internal and external applicants.

**Fig 2:**
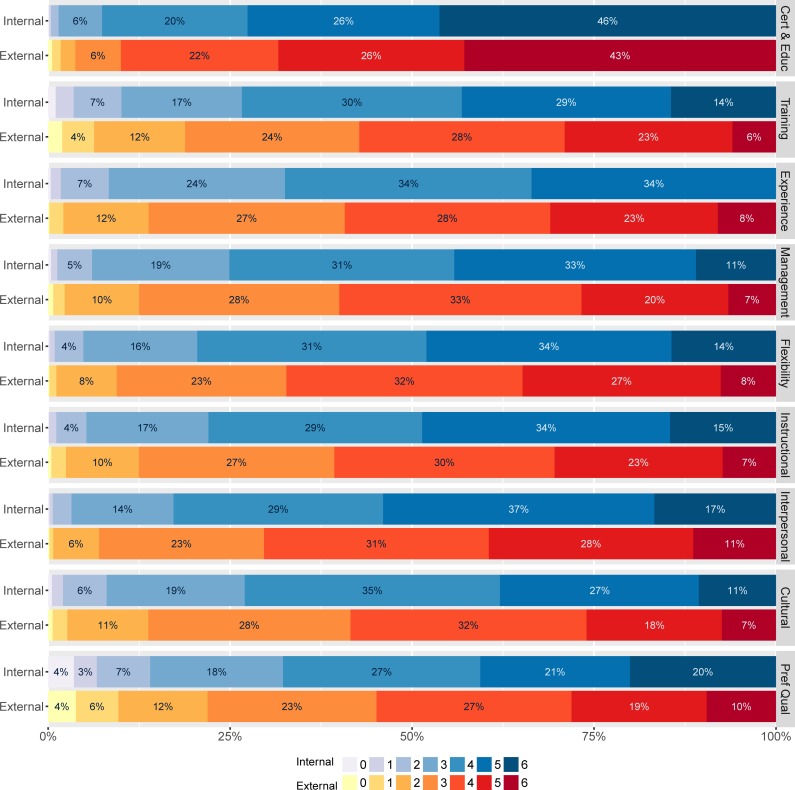
Distribution of subcomponent ratings for internal and external applicants.

Table 2, Fig 1 and Fig 2 demonstrate differences in rating of applicants internal and external to the district. While internal applicants’ total score is on average 39 points, external applicants score on average more than 3 points lower. Ratings are significantly lower for external applicants across all subcomponents.

Summative ratings of internal applicants remain significantly higher, by about 3 points, even when accounting for measures of teacher qualifications: previous teaching experience or state licensure scores (WEST-B). The difference is more apparent (around 4 points) when accounting for subsequent teacher quality estimated as teacher value added in subsample of applicants hired to Washington state (Table 3). These differences are consistent in all subsamples (S1 Table).

**Table 3:** Mixed effect models for summative total score.

	Model A	Model B	Model C	Model D1	Model D2	Model D
	Internal Only	Experience Only	WEST-B	VA Math Only	VA Read Only	Both VA
	N = 3474	N = 3473	N = 1411	N = 303	N = 336	N = 267
**Fixed Effects**	**Est (SE)**	**Est (SE)**	**Est (SE)**	**Est (SE)**	**Est (SE)**	**Est (SE)**
Intercept	36.03***	35.57***	36.23***	37.34***	36.96***	36.74***
	(0.48)	(0.50)	(0.60)	(1.32)	(1.11)	(1.37)
Internal	3.09***	3.16***	2.84***	3.97**	4.15***	4.80***
	(0.31)	(0.31)	(0.50)	(1.29)	(1.11)	(1.35)
Experience	-	0.11	-	-	-	-
		(0.03)				
WEST-B						
Writing	-	-	0.11	-	-	-
			(0.35)			
Reading	-	-	0.40	-	-	-
			(0.33)			
Math	-	-	0.09	-	-	-
			(0.27)			
Later VA						
Math	-	-	-	3.9	-	5.62*
				(2.00)		(2.46)
Reading	-	-	-	-	3.29	-3.10
					(2.27)	(3.04)
**Random Effects**	**Var (SD)**	**Var (SD)**	**Var (SD)**	**Var (SD)**	**Var (SD)**	**Var (SD)**
Appl:Sch	15.52	15.58	16.43	13.33	12.50	10.64
	(3.94)	(3.95)	(4.05)	(3.65)	(3.54)	(3.26)
Appl	10.22	10.26	5.16	4.97	5.37	3.75
	(3.20)	(3.20)	(2.27)	(2.23)	(2.32)	(1.94)
Rtr	12.07	11.96	11.25	10.42	11.14	12.21
	(3.50)	(3.46)	(3.35)	(2.23)	(3.34)	(3.49)
Sch	2.24	2.15	2.26	1.20	0.00	0.00
	(1.50)	(1.47)	(1.50)	(1.10)	(0.00)	(0.00)
Residual	21.15	20.95	21.28	14.07	15.85	15.71
	(4.60)	(4.58)	(4.61)	(3.75)	(3.98)	(3.96)

Notes: WEST-B: scores on state licensure test, standardized statewide, VA: teacher value added estimates, significance levels for p values

* p < 0.05

** p < 0.01

*** p < 0.001.

### Differences in variance decomposition and inter-rater reliability

**Fig 3:**
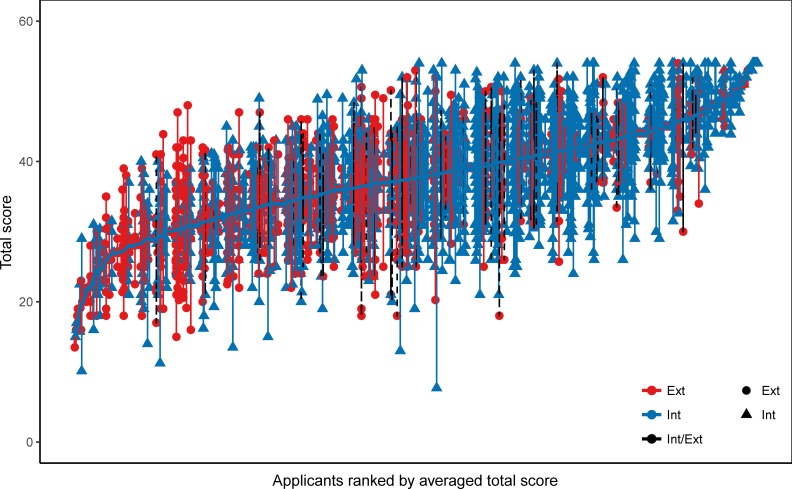
Mean and range of summative ratings of applicants rated multiple times between 2009–2013. Each vertical line connects summative ratings given to single applicant during this period. Applicants are ordered by average summative rating (solid circles).

Besides differences in ratings of external and internal applicants, we now pay attention to differences in precision of the ratings between the two groups (for summative score, see Fig 3).

**Fig 4:**
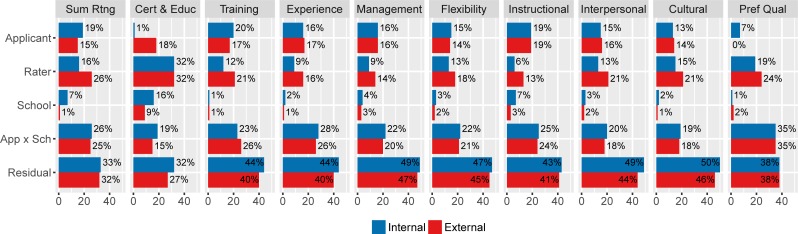
Variance decomposition for internal and external applicants calculated using Model (3) jointly on all data.

To assess differences in IRR between internal and external applicants, we provide decomposition of variance terms in joint Model (3) by applicant type, internal and external (Fig 4, S2 Table). We also provide comparison with a stratified approach, e.g. in [27, 28] (S3 Table). We observe that for the summative score, as well as for most of the subcomponents, *rater* variance is higher for external applications, i.e. ratings are less homogeneous when rating external applicants. In addition to higher rater variance, we also observe lower *applicant* variance for external applicants, i.e., external applicants (their qualities) are more homogeneous.

**Fig 5:**
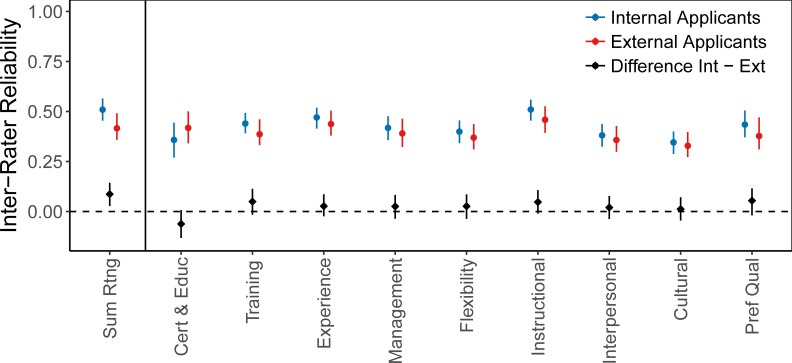
Within-school IRR estimates for internal applicants, external applicants and their difference, including bootstrap confidence intervals, calculated using Model (3) jointly on all data.

These differences in variance components result in lower IRR in external applicants (0.42, CI 0.36–0.49) than in internal applicants (0.51, CI 0.45–0.57), with the difference between internal and external IRR being significantly nonzero for summative scores (0.09, CI 0.03–0.14), see Fig 5 and S2 Table. The differences between internal and external IRR are confirmed as statistically significant by likelihood ratio tests. We find that Model (3) allowing for different variance terms in ratings of internal and external applicants fits significantly better than Model (1) for summative score as well as for subcomponents.

Note, if Model (1) is utilized for internal and external applicants separately (S3 Table), we also obtain higher rater variance and lower IRR for external applicants. However, this model does not allow for testing the significance of the difference, nor does it allow for different variance components in groups, or simultaneous use of information from applicants who were external in some applications but internal in others. Finally, Model (3) is more flexible in allowing the researcher to decide which variance components are treated as common for the two groups.

### Effect of higher number of raters on reliability and validity of scoring

Table 4 provides IRR estimates for the three scoring designs (using one, two and three raters per school). While the rule-of-thumb lower limit of 0.7 for reliability [34] can be reached for the summative score when the average of three raters are used for internal applicants, this 0.7 standard is not reached for external applicants.

**Table 4:** Effect of number of raters on reliability, standard error and predictive validity of scoring.

		Within-school IRR			Standard error of measures (SEM)			Estimated correlation with VA			
		1 rater	2 raters	3 raters	1 rater	2 raters	3 raters	1 rater	2 raters	3 raters	SEM = 0
**Summative rating**		** **	** **	** **	** **	** **	** **	** **	** **	** **	** **
	Internal	0.51	0.67	0.76	5.46	4.44	3.84	0.17**	0.19***	0.20***	0.23***
	External	0.42	0.59	0.68	6.05	5.08	4.47	0.17**	0.20***	0.21***	0.26***
**Cert. and Education**											
	Internal	0.36	0.53	0.63	0.87	0.75	0.66	0.01	0.02	0.02	0.02
	External	0.42	0.59	0.68	0.91	0.77	0.68	0.01	0.02	0.02	0.02
**Training**											
	Internal	0.44	0.61	0.70	0.96	0.80	0.70	0.09	0.11	0.12*	0.14*
	External	0.39	0.56	0.65	1.06	0.90	0.79	0.09	0.11	0.12*	0.15*
**Experience**											
	Internal	0.47	0.64	0.73	0.86	0.71	0.62	0.11	0.13*	0.14*	0.16*
	External	0.44	0.61	0.70	0.93	0.77	0.68	0.11	0.13*	0.14*	0.17*
**Management**											
	Internal	0.42	0.59	0.68	0.87	0.73	0.64	0.19***	0.23***	0.24***	0.30***
	External	0.39	0.56	0.66	0.91	0.77	0.68	0.19***	0.23***	0.25***	0.31***
**Flexibility**											
	Internal	0.40	0.57	0.67	0.86	0.73	0.64	0.13*	0.16**	0.17**	0.21***
	External	0.37	0.54	0.64	0.90	0.77	0.68	0.13*	0.16**	0.17**	0.22***
**Instructional Skills**											
	Internal	0.51	0.68	0.76	0.80	0.65	0.56	0.22***	0.25***	0.27***	0.31***
	External	0.46	0.63	0.72	0.86	0.72	0.62	0.22***	0.26***	0.28***	0.33***
**Interpersonal Skills**											
	Internal	0.38	0.55	0.65	0.84	0.72	0.64	0.14*	0.17**	0.19***	0.23***
	External	0.36	0.53	0.63	0.91	0.78	0.69	0.14*	0.17**	0.19***	0.24***
**Cultural Competency**											
	Internal	0.35	0.51	0.61	0.95	0.82	0.73	0.11	0.14*	0.15*	0.19***
	External	0.33	0.49	0.59	1.01	0.87	0.78	0.11	0.14*	0.15*	0.19***
**Prefer. Qualifications**											
	Internal	0.43	0.61	0.70	1.16	0.97	0.85	0.08	0.10	0.10	0.12*
	External	0.38	0.55	0.65	1.21	1.03	0.91	0.08	0.10	0.11	0.13*

We also find that for both the summative and subcomponent scores, the standard errors are quite large if only a single rater is employed for rating application materials (Table 4). For the summative score, standard error of measures (SEM) is over 5.0 which implies that the scores could easily move 10 points up or down, a very large gap relative to the 54-point scale. Across most subcomponents, SEM is higher for external applicants. Increasing the number of raters reduces the SEMs but differences between internal and external applicants in SEM remain large.

To summarize, using higher number of raters remarkably improves predictive validity (Table 4). In our case, predictive validity of the summative score for predicting subsequent teacher value added in math is estimated to increase from 0.17 to about 0.20 (an increase of 18%) for internal applicants when three raters are employed compared to a single rater. This increase is slightly higher for external applicants. Additionally, some subcomponents in cases of single ratings with insignificant correlations with value added (namely for Training, Experience, Cultural Competency, and Preferred Qualifications) are found to have significant correlations with value added with a higher number of raters (see Table 4).

## Discussion and conclusions

This study compared ratings for external and internal applicants to teacher positions. We find that in all subcomponents, insider applicants are rated higher than applicants without previous teaching experience or training in the district they are applying to work in. Notably, the difference in ratings remains significant even when accounting for various measures of applicant qualifications and quality. We also found that the reliability of ratings is significantly higher for internal applicants.

There are several possible explanations for lower and less precise ratings of external applicants. Many of the recommendations, upon which the ratings are based, for internal applicants are likely to come from employees in SPS who are familiar with the context and type of teachers the district seeks to hire. Thus, internal applicants are likely to have letter of recommendation writers who have good information about what the district is looking for, meaning some criteria may not be addressed in letters supporting external applicants, causing lower and less homogeneous ratings in external applicants. More information on rating criteria, and better prompts in terms of the kinds of information that the district is trying to illicit about teacher applicants may help reliably identify high-quality applicants from outside the district.

Additionally, raters may score an applicant higher and more consistently whom they have themselves observed, or an applicant whose letter of recommendation comes from a writer the rater knows personally. On the contrary, lower and more conservative ratings may be given to external candidates whose letter of recommendation comes from writers raters don’t know. Enabling the external candidates to volunteer or work for the district to obtain a letter of recommendation from district employees may thus help in this aspect.

As we have shown, higher number of raters may also help to increase reliability, decrease error variance and improve predictive power of applicant ratings. A higher number of raters might therefore be considered for rating external applicants to reach IRR levels comparable to those in internal applicants. Nevertheless, while higher number of raters has the potential to increase reliability of ratings, it is unlikely to solve the issue of lower, more conservative ratings of applicants from outside the district.

It is also worth pointing out methodological innovations used in this study that may be useful in other contexts. Specifically, to test group differences in inconsistencies in ratings, we employed model-based estimates of IRR. We have implemented the mixed-effect models to allow for analysis of IRR with unbalanced hierarchical structure of the data and we have allowed for different variance terms for different applicant status–a covariate which may moderate IRR. This approach was shown to be more flexible than stratifying data with respect to applicant status and estimating IRR separately for the two groups. Our model-based approach was able to more precisely describe the data, to jointly use information from the whole dataset and to detect differences in IRR between the two groups in cases when stratified analysis was not able.

Although we focus on applicant status (internal vs. external) as a moderator of IRR in the context of teacher hiring, this is just one example of a possible application of model-based IRR. IRR has been analyzed and compared for groups with respect to assessee or rater characteristics in journal peer review [35], grant peer-review [5, 28, 36], classroom observations of teachers [37–38], university candidates [3], student ratings, etc. In these areas and others, potential exists for assessee covariates, such as gender and ethnicity, rater characteristics such as rater position, experience or training [39–40] or covariates of units, e.g. school type or job type, which may moderate IRR and precision of ratings. In these cases, our model-based IRR may be able to detect differences in reliability between groups even when stratified IRR calculated separately for groups is not.

### Limitations

This paper investigates differences of ratings between internal and external applicants only on one of the stages of SPS selection process. However, other stages of the hiring process, e.g. the district-level rating or the interview stage may also introduce bias.

To explain the bias in ratings, we only examine three measures of teacher qualifications and quality. While being important predictors of teacher quality and student achievement [41–43], these measures are somewhat limited in how well they describe teacher quality. In particular, as we described above, one possible explanation for what appears to be bias in the ratings is that there is better social fit for internal applicants, i.e. that there is an unobserved factor influencing the internal-external differences. To investigate more thoroughly whether SPS might be losing high quality external applicants due to rating biases or to find evidence explaining why ratings of external applicants are lower, we would need other measures of teacher quality that may capture dimensions of teacher quality unaccounted for here, such as teacher observation scores, or student/family survey ratings.

Finally, there are additional complexities that might be addressed in future work. For example, our analysis treated the ratings as if they were all completed at the same time, however, some repeated ratings occurred in timespan of 5 years and applicant characteristics might have changed during this period.

### Conclusion

In conclusion, our study demonstrated lower and less precise ratings for external applicants to teacher positions with bias in ratings significant even when accounting for various measures of teacher qualifications and quality. This result is of high importance for educational research as well as for other fields, suggesting that high quality applicants who are “external” and have less connections to the institution and raters may be lost due to lower and less precise rating. As a result, the external applicants may be advised to become “insiders” before submitting an application, e.g. through volunteering, visits, substitute teacher or visiting positions, whenever possible. The institutions, on the other hand, might consider providing clearer guidance about what they are seeking when hiring, with a particular eye toward guidance aimed at applicants, and those recommending them, who do not know the district well.

Given the high stakes involved in ratings in many situations—e.g., ratings of job candidates, grant applications, journal submissions etc., we recommend investing resources to study and improve rating systems for ameliorating rating biases and inconsistencies across applicant subgroups.

## Supporting information

S1 Table Model 1A from Table 3 for restricted samples. (PDF)

S2 Table Decomposition of variance terms using Model (3) jointly for data of internal and external applicants. (PDF)

S3 Table Decomposition of variance terms when using Model (1) separately for internal and external applicants. (PDF)

S1 File Sample R code. (R)
