# Supplementary material for: Disparities in ratings of internal and external applicants: A case for model-based inter-rater reliability
Source: PLoS One. 2018 Oct 5;13(10):e0203002. doi: 10.1371/journal.pone.0203002 (PMC6173388; doi:10.1371/journal.pone.0203002)
Supplement: S1 Table — (PDF) [file pone.0203002.s001.pdf]

S1 Table. Model A from Table 3 for restricted samples.

Model	Unrestricted N = 3474	Model B Sample N = 3473	Model C Sample N = 1411	Model D Sample N = 267
**Fixed Effects**	**Est (SD)**	**Est (SD)**	**Est (SD)**	**Est (SD)**
Intercept	36.03***	36.06***	36.12***	36.61***
	(0.48)	(0.48)	(0.59)	(1.39)
Internal	3.09***	3.05***	2.89***	4.86***
	(0.31)	(0.32)	(0.49)	(1.36)
**Random Effects**	**Var (SD)**	**Var (SD)**	**Var (SD)**	**Var (SD)**
Appl:School	15.52	15.70	16.44	13.10
	(3.94)	(4.00)	(4.05)	(3.36)
Applicant	10.22	10.32	5.17	4.01
	(3.20)	(3.21)	(2.27)	(2.00)
Rater	12.07	12.05	11.34	12.53
	(3.47)	(3.47)	(3.37)	(3.54)
School	2.24	2.24	2.02	0.00
	(1.50)	(1.50)	(1.42)	(0.00)
Residual	21.15	20.93	21.31	15.36
	(4.60)	(4.58)	(4.62)	(3.92)
