# Supplementary material for: Disparities in ratings of internal and external applicants: A case for model-based inter-rater reliability
Source: PLoS One. 2018 Oct 5;13(10):e0203002. doi: 10.1371/journal.pone.0203002 (PMC6173388; doi:10.1371/journal.pone.0203002)
Supplement: S2 Table — (PDF) [file pone.0203002.s002.pdf]

S2 Table. Decomposition of variance terms using Model (3) jointly for data of internal and external applicants.

	Applicant type		Percentage of total variability					Total variability	Inter-rater reliability			IRR Difference Internal - External		
	b	SE(b)	Applicant	Rater	School	Appl:School	Residual		IRR Est.	LCI	UCI	Dif. Est.	LCI	UCI
**Summative rating**														
internal	3.34	0.40	19%	16%	7%	26%	33%	60.69	0.51	0.45	0.57	0.09	0.03	0.14
external			15%	26%	1%	25%	32%	62.67	0.42	0.36	0.49			
**Cert. and Education**														
internal	0.13	0.05	1%	32%	16%	19%	32%	1.18	0.36	0.27	0.44	-0.06	-0.13	0.01
external			18%	32%	9%	15%	27%	1.44	0.42	0.34	0.50			
**Training**														
internal	0.49	0.08	20%	12%	1%	23%	44%	1.65	0.44	0.39	0.49	0.05	-0.02	0.11
external			17%	21%	1%	21%	40%	1.82	0.39	0.33	0.46			
**Experience**														
internal	0.33	0.06	16%	9%	2%	28%	44%	1.40	0.47	0.41	0.52	0.03	-0.02	0.09
external			17%	16%	1%	26%	40%	1.53	0.44	0.38	0.50			
**Management**														
internal	0.41	0.06	16%	9%	4%	22%	49%	1.29	0.42	0.36	0.48	0.03	-0.04	0.08
external			16%	14%	3%	20%	47%	1.37	0.39	0.32	0.46			
**Flexibility**														
internal	0.35	0.05	15%	13%	3%	22%	47%	1.23	0.40	0.34	0.46	0.03	-0.04	0.09
external			14%	18%	2%	21%	45%	1.28	0.37	0.31	0.44			
**Instructional**														
internal	0.47	0.06	19%	6%	7%	25%	43%	1.31	0.51	0.45	0.56	0.05	-0.01	0.11
external			19%	13%	3%	24%	41%	1.38	0.46	0.39	0.53			
**Interpersonal**														
internal	0.31	0.05	15%	13%	3%	20%	49%	1.15	0.38	0.32	0.44	0.02	-0.04	0.08
external			16%	21%	2%	18%	44%	1.29	0.36	0.30	0.43			
**Cultural**														
internal	0.34	0.05	13%	15%	2%	19%	50%	1.39	0.35	0.29	0.40	0.01	-0.05	0.07
external			14%	21%	1%	18%	46%	1.51	0.33	0.27	0.40			
**Preferred Qualifications**														
internal	0.47	0.09	7%	19%	1%	35%	38%	2.37	0.43	0.37	0.50	0.05	-0.02	0.12
external			0%	24%	2%	35%	38%	2.34	0.38	0.31	0.47			
