# Supplementary material for: Disparities in ratings of internal and external applicants: A case for model-based inter-rater reliability
Source: PLoS One. 2018 Oct 5;13(10):e0203002. doi: 10.1371/journal.pone.0203002 (PMC6173388; doi:10.1371/journal.pone.0203002)
Supplement: S3 Table — (PDF) [file pone.0203002.s003.pdf]

**S3 Table. Decomposition of variance terms when using Model (1) separately for internal and external applicants.**

| | Percentage of total variability | | | | | Total variability | Inter-rater reliability | | |
|---|---|---|---|---|---|---|---|---|---|
| | Applicant | Rater | School | Appl:School | Residual | | IRR Est. | LCI | UCI |
| **Internal** | | | | | | | | | |
| Summative Rating | 17% | 17% | 5% | 30% | 31% | 60.80 | 0.52 | 0.46 | 0.58 |
| Certificate and Education | 2% | 35% | 12% | 21% | 31% | 1.14 | 0.34 | 0.26 | 0.43 |
| Training | 17% | 13% | 0% | 27% | 43% | 1.67 | 0.44 | 0.39 | 0.51 |
| Experience | 15% | 9% | 3% | 30% | 43% | 1.41 | 0.48 | 0.42 | 0.54 |
| Management | 15% | 9% | 3% | 26% | 46% | 1.27 | 0.45 | 0.39 | 0.51 |
| Flexibility | 15% | 13% | 3% | 25% | 44% | 1.22 | 0.42 | 0.36 | 0.49 |
| Instructional | 20% | 6% | 6% | 28% | 39% | 1.29 | 0.54 | 0.48 | 0.60 |
| Interpersonal | 16% | 12% | 3% | 23% | 47% | 1.13 | 0.41 | 0.35 | 0.47 |
| Cultural | 12% | 15% | 2% | 25% | 47% | 1.37 | 0.38 | 0.32 | 0.44 |
| Preferred Qualifications | 6% | 19% | 0% | 41% | 33% | 2.38 | 0.47 | 0.41 | 0.55 |
| **External** | | | | | | | | | |
| Summative Rating | 17% | 26% | 3% | 20% | 34% | 61.74 | 0.41 | 0.32 | 0.49 |
| Certificate and Education | 15% | 30% | 8% | 14% | 33% | 1.41 | 0.37 | 0.27 | 0.47 |
| Training | 19% | 23% | 0% | 20% | 38% | 1.78 | 0.39 | 0.31 | 0.48 |
| Experience | 19% | 16% | 0% | 26% | 39% | 1.47 | 0.44 | 0.37 | 0.53 |
| Management | 15% | 12% | 6% | 15% | 51% | 1.40 | 0.36 | 0.26 | 0.46 |
| Flexibility | 14% | 17% | 4% | 16% | 48% | 1.30 | 0.35 | 0.25 | 0.45 |
| Instructional | 16% | 11% | 5% | 21% | 48% | 1.43 | 0.42 | 0.32 | 0.51 |
| Interpersonal | 13% | 16% | 6% | 19% | 45% | 1.31 | 0.39 | 0.29 | 0.48 |
| Cultural | 15% | 19% | 0% | 11% | 55% | 1.49 | 0.26 | 0.17 | 0.36 |
| Preferred Qualifications | 0% | 23% | 4% | 28% | 45% | 2.32 | 0.32 | 0.20 | 0.45 |
